# Radiation-induced DNA double-strand breaks in cortisol exposed fibroblasts as quantified with the novel foci-integrated damage complexity score (FIDCS)

**DOI:** 10.1038/s41598-024-60912-y

**Published:** 2024-05-06

**Authors:** Wilhelmina E. Radstake, Alessio Parisi, Silvana Miranda, Kiran Gautam, Randy Vermeesen, Emil Rehnberg, Kevin Tabury, Rob Coppes, Marc-Jan van Goethem, Sytze Brandenburg, Ulrich Weber, Claudia Fournier, Marco Durante, Bjorn Baselet, Sarah Baatout

**Affiliations:** 1 Radiobiology Unit, Belgian Nuclear Research Centre (SCK CEN), Mol, Belgium; 2 Department of Molecular Biotechnology, Faculty of Bioscience Engineering, Ghent University, Ghent, Belgium; 3 Radiation Protection Dosimetry and Calibration Expert Group, Belgian Nuclear Research Centre (SCK CEN), Mol, Belgium; 4 Department of Radiation Oncology, Mayo Clinic, Jacksonville, FL USA; 5 Department of Biomedical Engineering, University of South Carolina, Columbia, USA; 6 Department of Biomedical Sciences of Cells and Systems, Section of Molecular Cell Biology, University of Groningen, University Medical Center Groningen, 9713 Groningen, The Netherlands; 7 Department of Radiation Oncology and Particle Therapy Research Center, University Medical Center Groningen, University of Groningen, Groningen, The Netherlands; 8 Biophysics Division, GSI Helmholtzzentrum Für Schwerionenforschung GmbH, Darmstadt, Germany; 9 Institute for Condensed Matter Physics, Technische Universität Darmstadt, Darmstadt, Germany

**Keywords:** Ionizing radiation, Iron ions, Carbon ions, Protons, Cortisol, Fibroblast, DNA damage, DNA repair, Fluorescence imaging, Double-strand DNA breaks

## Abstract

Without the protective shielding of Earth’s atmosphere, astronauts face higher doses of ionizing radiation in space, causing serious health concerns. Highly charged and high energy (HZE) particles are particularly effective in causing complex and difficult-to-repair DNA double-strand breaks compared to low linear energy transfer. Additionally, chronic cortisol exposure during spaceflight raises further concerns, although its specific impact on DNA damage and repair remains unknown. This study explorers the effect of different radiation qualities (photons, protons, carbon, and iron ions) on the DNA damage and repair of cortisol-conditioned primary human dermal fibroblasts. Besides, we introduce a new measure, the Foci-Integrated Damage Complexity Score (FIDCS), to assess DNA damage complexity by analyzing focus area and fluorescent intensity. Our results show that the FIDCS captured the DNA damage induced by different radiation qualities better than counting the number of foci, as traditionally done. Besides, using this measure, we were able to identify differences in DNA damage between cortisol-exposed cells and controls. This suggests that, besides measuring the total number of foci, considering the complexity of the DNA damage by means of the FIDCS can provide additional and, in our case, improved information when comparing different radiation qualities.

## Introduction

In space, astronauts are exposed to higher levels of ionizing radiation than on Earth. Different sources contribute to this cosmic radiation field, including the Sun, trapped particles in the Van Allen Belts, and galactic cosmic rays^1^. The latter consists of highly charged, high energy nuclei (HZE), which are thought to derive from supernovas outside our solar system and provide a continuous background of high linear energy transfer (LET) radiation. Due to their high energy, HZE nuclei are very difficult to stop. Furthermore, the biological effects of exposure to these particles are mostly stochastic and increased exposure to this type of high-LET radiation is associated with several health risks. For this reason, radiation is considered the main showstopper for manned deep space exploration^2^.

When ionizing radiation traverses the cell nucleus, it induces breakage and disruption of the DNA strands^3^. High-LET particles are highly ionizing along their track and are therefore more efficient in inducing DNA double-strand breaks (DSBs). Additionally, the cells’ ability to repair DNA damage becomes compromised after exposure to high-LET cosmic radiation, as this type of radiation increases the amount of complex DNA damage, defined as more than two lesions within two helical turns^4,5^.

The formation of DNA DSBs leads to an arrest in cell cycle division and DNA damage repair pathways are initiated. Within minutes after DNA damage induction, protein modification such as phosphorylation of histone H2AX (γ-H2AX) appear near the damaged side^6,7^. Furthermore, proteins are recruited to the side of the breakage and localize in radiation-induced foci (RIF). One of such proteins is p53 binding protein 1 (53BP1), which localizes within a few minutes after induction of DNA DSBs^8^. High levels of DNA DSBs can lead to cellular apoptosis. However, in cases of damage repair, the surviving cell can still become carcinogenic as a result of chromosomal aberrations or translocations^9^.

Besides higher levels of ionizing radiation, spaceflight-related stressors such as social isolation, living in a confined environment, and a high workload can contribute to an increase in psychological stress. During stressful events, the hypothalamic-pituitary-adrenocortical (HPA) axis is activated, which leads to an increase in circulating glucocorticoids. Acute stress is characterized by a temporary rise in cortisol levels that return to baseline levels after the stressful event is eliminated. However, in cases of sustained stress, the function of the HPA axis becomes dysregulated, which leads to chronic high levels of circulating glucocorticoids^10^. This type of sustained stress is considered an important risk factor for the development of diseases related to autonomic, cardiovascular, gastrointestinal, and immune system dysfunction^11^.

Increased levels of cortisol have been measured in astronauts after short- and long-term spaceflight^12–15^. Higher levels of cortisol in blood plasma have been linked to a suppression of DNA repair capacity^16^. Besides, fibroblasts that were exposed to cortisol in the culture medium showed an increased incidence of DNA strand breaks compared to unexposed cells. Moreover, the ability to repair this DNA damage induced by exposure to ultraviolet light was also impaired in these cells^17^.

It is currently unknown whether and how exposure to cortisol may affect the repair process of fibroblasts exposed to different qualities of ionizing radiation. Therefore, in this work, we exposed primary human dermal fibroblasts to different qualities of ionizing radiation, including photons, protons, carbon, and iron ions, to investigate the induction and repair of DNA DSBs. Additionally, cells were exposed to cortisol to investigate the effects of this stress hormone on the DNA repair process.

The DNA damage was measured with fluorescent microscopy, a commonly used methodology that quantifies DNA damage by counting the number of RIF. However, especially with HZE particle radiation, such quantification becomes challenging as DNA DSBs are in close proximity to each other and difficult to distinguish due to foci overlap. This issue can be overcome by using advanced super resolution and atomic force microscopes, which are able to image finer and more detailed structures^18,19^. However, such microscopes are expensive, not commonly available, and have limited throughput with respect to conventional microscopes. Therefore, we introduce a new measure called the Foci Integrated Damage Complexity Score (FIDCS), which provides a measure of DNA damage based on foci parameters that can be quantified with more conventional fluorescent microscopes, namely the focus area and its fluorescent intensity. The DNA damage assessed with this novel FIDCS is compared to corresponding experimental estimations using the number of foci and to the results of nanoscale computer simulations.

## Materials and methods

### Fibroblast culture

Primary normal human dermal fibroblasts (NHDF, PromoCell, C-12302), originating from one donor (33-year-old Caucasian female), were cultured at 37 °C and 5% CO_2_ in Dulbecco’s Modified Eagle Medium with GlutaMAX (DMEM, Gibco, 10566016). The medium was supplemented with 10% fetal bovine serum (FBS, Gibco, 10500064) and 0.25% Penicillin–Streptomycin (Pen-Strep, Sigma-Aldrich, P4333). Passage of cells occurred at 80–90% confluence using 0.05% Trypsin–EDTA (Gibco, 25300062). The experiments were performed with asynchronized cells with passage numbers between 4 and 7.

### Experimental procedures

Asynchronized exponentially growing cells were seeded inside IBIDI 96 microwell plates (89626) at densities of 3,000 cells per well (0.5 cm^2^) using full serum DMEM and left to attach overnight. Afterward, cells were washed with phosphate buffered saline (PBS) and incubated with DMEM containing hydrocortisone (HC, Sigma-Aldrich, H0888) at a concentration of 1 µmol/L or with a control vehicle. Hydrocortisone was first diluted in 96% ethanol at a concentration of 1 mg/ml. This was then further diluted in PBS to obtain a stock solution of 100 µmol/L which was again diluted in the cell culture media. For the control vehicle, these dilution steps were repeated without the addition of hydrocortisone. After 48 h of incubation with stress hormones or a control vehicle, cells were exposed to different radiation qualities (see section "Exposure to ionizing radiation") at doses of 0.1, 0.5, and 1 Gy or sham irradiated. At the time of irradiation, the cultures were reaching confluency. After irradiation, cells were left to incubate at 37 °C for 30 min, 1 h, 4 h or 48 h. Afterwards, cells were rinsed with PBS, fixed using a 10% Formalin solution (Sigma-Aldrich, HT5014), and stored in PBS at 4 °C until further manipulation.

### Exposure to ionizing radiation

The irradiations with different radiation qualities, including photons, protons, carbon, and iron ions, were carried out at different radiation facilities in Europe. Table 1 provides an overview of the radiation qualities, mean linear energy transfer (LET) values, and microdosimetric quantities for each radiation exposure. These quantities were obtained by means of computer simulations with PHITS^20^, which are detailed in the Supplementary Materials. Irradiations were performed at room temperature.

**Table 1:** Overview of the radiation quality, energy, LET, and microdosimetric quantities.

Radiation type	Energy	LET _D_, _primary_ (keV/µm)	LET _D, all_ (keV/µm)	y _D_ (keV/µm)
^137^Cs γ-rays	662 keV	–	0.3	2.2
^1^H ions	150 MeV	0.56	3.8	5.0
^12^C ions	90 MeV/n	28.2	29.3	18.1
^56^Fe ions	1000 MeV/n	155	155	73.1

LET_D, primary_ = unrestricted dose-mean linear energy transfer (LET) in water of the primary beam (without fragments). LET_D, all_ = unrestricted dose-mean linear energy transfer (LET) of all particles (including all secondary particles). y_D_ = dose-mean lineal energy in water (target: liquid water spheres with diameter equal to 0.6 µm).

The cells were exposed to γ-rays using an IBL 637 Cesium-137 source provided by the University Medical Center Groningen (UMCG), the Netherlands. The well-plates were placed on a Plexiglas plate in a horizontal position and irradiated from the bottom at approximately 0.008 Gy/sec. The exposures to protons (150 MeV) and carbon ions (90 MeV/n) were carried out at the UMCG Particle Therapy Research Center (PARTREC) facility in Groningen, the Netherlands. The well plates were completely filled with medium before irradiation, sterile sealed using Aeroseal membranes, and covered with sterile parafilm. The samples were irradiated in a vertical position, through the bottom of the well plate. The irradiations were performed with a continuous scanned broad beam with homogeneous fluence in the plateau of the Bragg curve. During proton irradiations, dose buildup was achieved using an 18 mm polycarbonate plate upstream of the cells. The dose-rate was approximately 0.5 Gy/min for both proton and carbon irradiations. The irradiations with iron ions (1 GeV/n) were carried out at the GSI-FAIR facility in Darmstadt, Germany. The well plates were sealed in a similar fashion as during proton and carbon ion exposure. The samples were irradiated through the bottom of the plate in a vertical position with a scanned pencil beam with homogeneous fluence in the plateau of the Bragg curve. For iron ions, 1 Gy corresponded to a fluence of 4 × 10^6^ ions/cm^2^. Cells were irradiated with 0.1, 0.5, or 1 Gy (+ /-−2% for protons, ~ 5% for carbon and iron ions).

### DNA double-strand breaks quantification

For each condition and at each time point (30 min, 1 h, 4 h, and 48 h), four wells were used as technical replicates for immunocytochemical visualization of DNA DSBs. Fixed cells were incubated in PBS containing 0.25% Triton X-100 for three minutes at room temperature. After rinsing with PBS, samples were blocked with Tris-NaCl-blocking buffer (TNB) containing 5% goat serum for one hour at room temperature. Primary antibodies (mouse monoclonal to γ-H2AX, Millipore 05–636, at 1/300 and rabbit polyclonal to 53BP1, Novus Biological NB 100–304, at 1/1000) were diluted in TNB and added to the samples for one hour at 37 °C. Samples were washed three times with PBS and incubated with TNB containing secondary antibodies (Alexa Fluor 488 goat-anti-mouse, 1/300 and Alexa Fluor 568 goat-anti-rabbit, 1/1000) for one hour at 37 °C. Cells were rinsed and mounted with IBIDI mounting medium containing DAPI (IBIDI, 50,011). A Nikon Ti Eclipse inverted wide field fluorescence microscope (Nikon Instruments) with a 20 × objective connected to a Prime BSI sCMOS camera was used to visualize cell nuclei, γ-H2AX and 53BP1 foci. Per well, images at four locations were obtained with z-stacks of 11 images that were taken 0.9 µm apart.

#### Image analysis

Fiji (v1.53C, https://fiji.sc) was used for image processing. All images were summed across the z-axis. The Fiji macro processing pipeline as described by De Vos et al.^21^ was used to quantify the number of cell nuclei, the number of foci, the area of each focus, and the intensity of each focus. Nuclei were segmented by thresholding DAPI images following the IJ-IsoData threshold algorithm and watershed. Spot segmentation was done by thresholding at a fixed threshold of 90 (software-defined arbitrary unity) for the γ-H2AX images and at 200 for the 53BP1 images. Foci smaller than 5 pixels were discarded.

#### Subtraction of the background DNA damage

For both techniques used in this study, namely the foci counting (paragraph “DNA damage quantification by foci counting”) and the FIDCS (paragraph “Foci-Integrated Damage Complexity Score (FIDCS)”), the baseline DNA damage was independently assessed for each timepoint by means of unirradiated control samples.

In a second step, the signal (foci or FIDCS) from the control samples (*S*_control_) was subtracted from the signal stemming from the experimental exposure (*S*_exp_) to obtain an estimate of the radiation-induced DNA damage (*S*_radiation_).

1 $$S_{radiation} = S_{exp} - S_{control} = \frac{{\mathop \sum \nolimits_{i = 1}^{{N_{wells \,\,exp} }} s_{well\,\, exp\,\, i} }}{{N_{wells \,\,exp} }} - \frac{{\mathop \sum \nolimits_{i = 1}^{{N_{wells \,\,control} }} s_{well \,\,control \,\,i} }}{{N_{wells \,\,control} }}$$

where $s_{{well\,\,{ }exp\,\,{ }i}}$ is the signal from one of the irradiated well ($N_{wells\,\,exp} = 4$) and $s_{{well{ }\, control{ }\, i}}$ is the signal from one of the control well ($N_{{wells{ }\,\,control}} = 4$).

#### DNA damage quantification by foci counting

In case of foci counting, the signal from one well (either irradiated or control) was assessed as

2 $$s_{well\,\, i\,\, foci\,\, counting} = \frac{{f_{well\, i} }}{{n_{well \,i} }}$$

where $f_{well\, i}$ is the number of foci in the well and $n_{well\, i}$ is the number of nuclei in the well. The analysis was carried out separately considering the γ-H2AX foci, the 53BP1 foci, and γ-H2AX-53BP1 colocalized foci.

#### Foci-integrated damage complexity score (FIDCS)

Under the assumption that the amount and complexity of DNA damage can be correlated with the amount of γ-H2AX and 53BP1 recruited at the location of the strands rupture, we hypothesized that the total luminescent signal in the cell nuclei is proportional with the number of DNA DSBs as in Eq. 3.

3 $$s_{well \,\,i \,\,FIDCS} = \frac{{\mathop \sum \nolimits_{j = 1}^{{j = f_{well \,i} }} \left( {A_{j \,well\, i} \cdot I_{j \,well\, i} } \right)}}{{n_{well\, i} }}$$

where $A_{j \,\, well \,\,i}$ is the area of the focus *j*, $I_{j \,\,well \,\,i}$ is the intensity of focus *j*, $f_{well \,\,i}$ is the number of foci in the well, and $n_{well\,\,i}$ is the number of nuclei in the well.

In other words, for each individual focus the area was multiplied by the intensity. These values were summed for all the foci within one well and divided by the number of nuclei in each well. For the above-mentioned measures, average values and standard deviations were calculated over the four wells per each condition. Within each well a minimum of 100 cells were imaged. These values were averaged over the four wells per condition. The analysis was carried out separately considering the γ-H2AX foci and 53BP1 foci.

### Statistical analysis

All statistical tests and data plotting were performed in R version 2022.07.0^22^. To investigate the effect of each condition, average values and standard deviation of the measures described above were calculated for four replicate wells.

First, the data were checked for outliers. Data points that laid 1.5 times the interquartile range above the upper quartile or below the lower quartile were removed from the data. The remaining values as described above were then used to plot the data as a function of dose.

For the statistical model, average, baseline-subtracted values of number of γ-H2AX, number of 53BP1, and number of colocalized foci, as well as γ-H2AX and 53BP1 FIDCS were used in a general linear regression model (assuming Gaussian distribution) to test for main and interaction effects of the independent variables stress, dose, and radiation quality (Table 2). As the number of RIF depends on time in a non-linear fashion, time was included as a categorical independent variable as well. For all linear regression models, the nuclear area was included as a covariate of non-interest. To guarantee unbiased estimates, residual errors were taken into account. Furthermore, Akaike information criterion (AIC) values were considered for the goodness-of-fit of the model. The notation for the different conditions as shown in the tables in the Supplementary Materials is provided in Table 2 as well. For the categorical variables of stress, radiation quality, and time, each level was compared to the control condition (control vehicle, photon radiation, and 30 min time point) and hence, these control conditions do not have a notation.

**Table 2:** Overview of experimental conditions and regression model variables.

Variable	Levels	Notation
Stress	Control vehicle	
	Cortisol	x_cort_
Dose	Continuous	x_d_
Radiation quality	Photons	
	Protons	x_prot_
	Carbon ions	x_C_
	Iron ions	x_Fe_
Time	30 min	
	1 h	x_t1_
	4 h	x_t4_
	48 h	x_t48_

## Results

### Number of DNA double-strand breaks after exposure to different radiation qualities and cortisol

**Figure 1:**
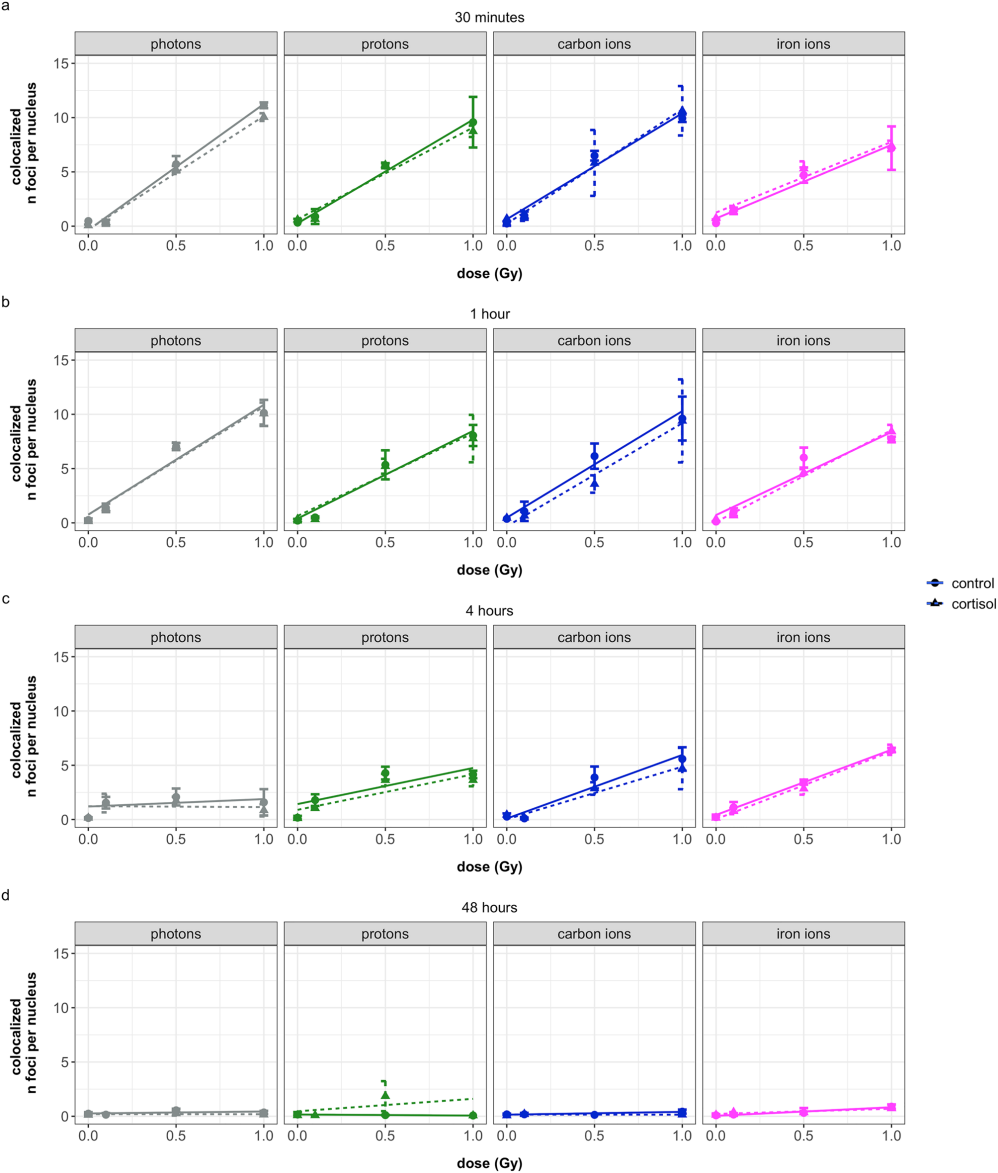
Overview of the number of colocalized foci at different time points after exposure of NHDF to different radiation qualities and with or without addition of cortisol (1 µmol/L). a: cell fixation at 30 min after irradiation, b: cell fixation at 1 h after irradiation, c: cell fixation at 4 h after irradiation, d: cell fixation at 48 h after irradiation. Photons = γ-rays. Plots show the mean (dots) with standard deviations (error bars) of four replicate wells, lines show linear regression lines (dotted line for cortisol). Values are baseline-subtracted. Four wells per condition, with an average of 440 nuclei per well were imaged. The corresponding graphs for the number of γ-H2AX and 53BP1 foci can be found in Supplementary Figures S2 and S3, respectively.

For each time point, the influence of dose, stress exposure and radiation quality on the number of γ-H2AX, 53BP1, and colocalized foci was determined (Supplementary Figures S2 and S3, and Fig. 1, respectively). Here we present the results related to the number of colocalized foci, for the analysis of the number of γ-H2AX and 53BP1 foci, readers are referred to the supplementary material. A three-way interaction between time, dose, and radiation quality was included in the model as well as a main effect of stress. Because the size of the nucleus constrains the number of RIF, the average nuclear size was included in the model as covariate of non-interest. Using these parameters, the model was significant (AIC = -40.01, R^2^ = 0.92, F(33, 471) = 174.4, *p* < 0.0001). The coefficients of significant estimates are shown in Supplementary Table S1. Table 3 shows the analysis of variance for the regression model. Both time and dose significantly affected the number of colocalized RIF. The number of RIF linearly increased with dose. However, this increase depended on the radiation quality (and thus LET) and for cells irradiated with iron ions, the increase in RIF with increasing dose was significantly lower at 30 min after irradiation, compared to photon irradiation at the same time point (Fig. 1c, Supplementary Table S1).

**Table 3:** ANOVA table for the regression model of average number colocalized RIF.

	Df	Sum Sq	F value	*p* value
Time	3	1088.5	419.17	< 0.0001
Stress	1	0.74	0.85	0.3571
Dose	1	2510.5	2900.32	< 0.0001
Radiation quality	3	2.82	1.08	0.3551
Nuclear area	1	7.81	9.02	0.0028
Time*dose	3	1151.36	443.38	< 0.0001
Time*radiation quality	9	77.87	10.00	< 0.0001
Dose*radiation quality	3	10.76	4.15	0.0065
Time*dose*radiation quality	9	132.21	16.97	< 0.0001
Residuals	471	407.69		

*Df* degree of freedom, *Sum Sq* sum of squares.

With increase in time, the linear relation between dose and number of RIF decreased, as observed by the flattening of the slope in the plots in Fig. 1. However, this effect depended on the radiation quality. The largest differences between the results of the different radiation qualities were observed four hours after the radiation exposure (Fig. 1c). At this time point, the cells irradiated with 1 Gy of photons had significantly lower number of colocalized RIF compared to other radiation qualities. The cells irradiated with 1 Gy of iron ions showed the highest number of colocalized RIF at this timepoint.

### Area and intensity of γ-H2AX and 53BP1 foci

**Figure 2:**
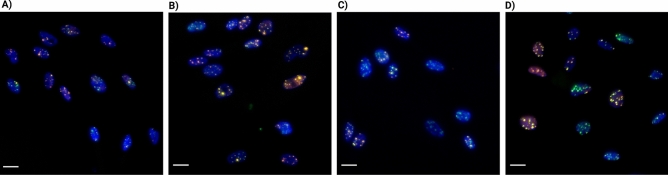
Overview of immunofluorescent staining of γ-H2AX (green), and 53BP1 (orange) for DNA double-strand breaks and DAPI for cell nuclei (blue). NHDF cells were fixed 30 min after irradiation, images show cells exposed to 1 Gy, without cortisol. (**A**) Photon irradiation (γ-rays using an IBL 637 Cesium-137 source). (**B**) Protons of 150 MeV. (**C**) Carbon ions at 90 MeV/n. (**D**) Iron ions at 1000 MeV/n. Scale bar = 20 µm. 0 Gy control sample staining shown in Supplementary Figure S1.

The results presented above provided insights into the influence of dose, stress exposure, and radiation quality on the number of colocalized RIF. However, visual examination of the data showed that the observed damage in high-LET radiation, particularly with iron ions, appeared as larger foci with higher intensity and was more substantial than what was captured solely by the number of RIF (Fig. 2).

To better understand the damage to the DNA based on radiation quality, we next analyzed each focus by multiplying the intensity by the area of both γ-H2AX and 53BP1 separately. These values were summed for all foci within each replicate well and divided by the number of nuclei in each well to obtain a measure of total DNA damage, or Foci-Integrated Damage Complexity Score (FIDCS), per well (see paragraph “Subtraction of the background DNA damage”–“Foci Integrated Damage Complexity Score (FIDCS)”). We like to point out that the method described in this paper is methodologically different to the one described by Roobol et al. who quantified the area and intensity of 53BP1 signal for the average focus^23^. Instead, we summed the product of area and intensity for each individual focus over the entire well which was then divided by the number of nuclei to evaluate the average damage within one nucleus. We then used linear regression to determine the effects of radiation quality, cortisol exposure, time, and dose on the FIDCS.

For γ-H2AX FIDCS, the model was fitted with four three-way interaction terms between (1) dose, stress, and time, (2) dose, stress, and radiation quality, (3) stress, time, and radiation quality, and (4) dose, time, and radiation quality. The nuclear area was included as a covariate of non-interest. The model was significant (AIC = 9770, R^2^ = 0.79, F(55, 442) = 30.85, *p* < 0.0001). Table 4 presents the ANOVA results of the regression model, which shows the variance in the data that is captured by the model. The coefficients of the regression model, showing the relationships and magnitudes of the predictors with the γ-H2AX FIDCS, are shown in Supplementary Table S6. γ-H2AX FIDCS linearly increased with dose, this effect however was different depending on the other factors of stress, radiation quality, and time.

**Table 4:** ANOVA table for the regression model of γ-H2AX FIDCS.

	Df	Sum Sq	F-value	*p* value
Nuclear area	1	1.15E + 09	3.86	0.0501
Dose	1	2.20E + 11	738.42	< 0.0001
Stress	1	4.72E + 09	15.83	< 0.0001
Time	3	4.60E + 10	51.44	< 0.0001
Radiation quality	3	2.88E + 10	32.23	< 0.0001
Dose*stress	1	3.48E + 09	11.66	0.0007
Dose*time	3	6.04E + 10	67.49	< 0.0001
Stress*time	3	3.03E + 09	3.39	0.0181
Dose*radiation quality	3	2.57E + 10	28.78	< 0.0001
Stress*radiation quality	3	3.04E + 09	3.39	0.0179
Time*radiation quality	9	5.74E + 10	21.40	< 0.0001
Dose*stress*time	3	2.35E + 09	2.63	0.0496
Dose*stress*radiation quality	3	1.75E + 09	1.96	0.1196
Stress*time*radiation quality	9	8.19E + 09	3.05	0.0015
Dose*time*radiation quality	9	3.97E + 10	14.80	< 0.0001
Residuals	442	1.32E + 11		

*Df* degree of freedom, *Sum Sq* sum of squares.

**Figure 3:**
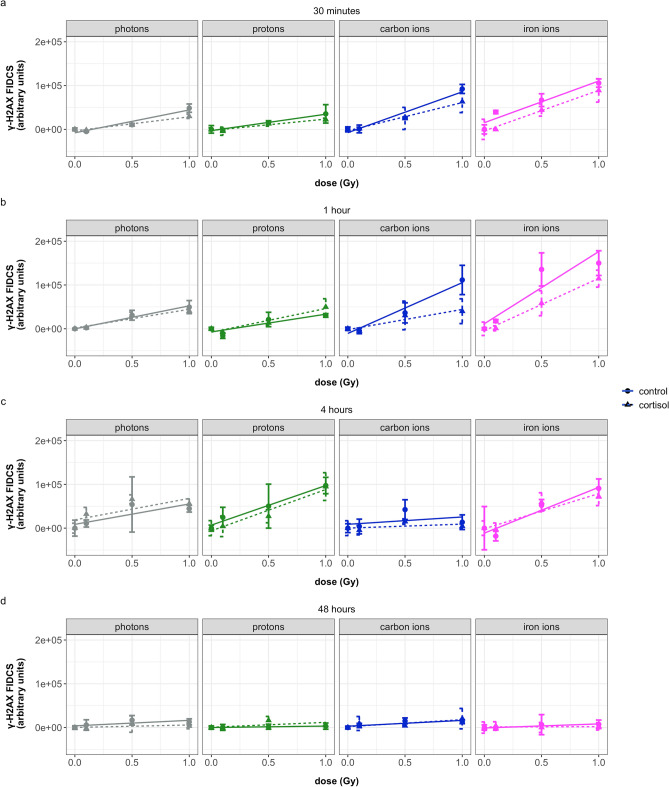
Overview of FIDCS for γ-H2AX at different time points after exposure of NHDF to different radiation qualities, and with or without addition of cortisol (1 µmol/L). (**a**) cell fixation at 30 min after irradiation, (**b**) cell fixation at 1 h after irradiation, (**c**) cell fixation at 4 h after irradiation, (**d**) cell fixation at 48 h after irradiation. Photons = γ-rays. FIDCS are calculated by multiplying the area by the intensity for each individual focus. Plots show mean (dots) with standard deviations (error bars) for four replicate wells, lines show linear regression lines (dotted lines for cortisol). Values are baseline subtracted. Four wells per condition, on average 440 nuclei per well were imaged.

After 30 min, in carbon and iron ion exposed cells, the linear increase with dose was significantly higher than photon and proton exposed cells (Fig. 3a). One hour after iron ion exposure, the increase in FIDCS as a function of dose was significantly higher in iron exposed cells compared to other radiation qualities. At four hours, cells exposed to protons, exhibited a significant increase in the slope for γ-H2AX FIDCS compared to other timepoints for proton exposed cells. At this timepoint, the slope for cells exposed to carbon ions was significantly lower than that of photon exposed cells (Fig. 3c). At 48 h, there was a significant decrease in the slope, indicating a possible repair of the DNA DSBs as the FIDCS did not significantly increase with dose at that timepoint (Fig. 3d).

In general, adding the overall effect of cortisol exposure to the regression model significantly improved the model’s performance, helping to better understand the variation in the data (as highlighted in Table 4). For most radiation qualities and timepoints, the increase in FIDCS with dose was lower in cells exposed to cortisol compared to cells exposed to the control vehicle. This effect was especially observed at 30 min and 1 h after carbon and iron ion exposure. Yet, when looking at the detailed results from our regression model (Supplementary Table S1), no significant coefficients were observed for the (interaction-)factors that included cortisol. This indicates that although cortisol exposure significantly explained a proportion of the variance in the data, its direct role on the γ-H2AX was not strong enough to stand out on its own and more data would be needed to explore the relationship between stress and the γ-H2AX FIDCS.

Next, the FIDCS for 53BP1 was investigated. The model was fitted with three three-way interaction terms between (1) dose, stress, and time, (2) radiation quality, stress, and time, and (3) radiation quality, dose, and time. The model was significant (AIC = 9991, R^2^ = 0.74, F(52, 457) = 25,35, *p* < 0.0001). The coefficients of the regression model are shown in Supplementary Table S7. The ANOVA table of the regression model is shown in Table 5.

**Table 5:** ANOVA table for the regression model of 53BP1 FIDCS.

	Df	Sum Sq	F value	*p* value
Dose	1	1.34E + 11	457.29	< 0.0001
Stress	1	2.36E + 09	8.09	0.0046
Time	3	9.08E + 10	103.70	< 0.0001
Radiation quality	3	2.06E + 10	23.48	< 0.0001
Nuclear area	1	1.24E + 10	42.35	< 0.0001
Dose*stress	1	6.64E + 08	2.28	0.1321
Dose*time	3	6.21E + 10	70.89	< 0.0001
Stress*time	3	4.26E + 09	4.86	0.0024
Stress*radiation quality	3	5.09E + 09	5.81	0.0007
Time*radiation quality	9	2.36E + 10	8.97	< 0.0001
Dose*radiation quality	3	1.16E + 10	13.22	< 0.0001
Dose*stress*time	3	2.58E + 09	2.94	0.0327
Stress*time*radiation quality	9	5.91E + 09	2.25	0.0182
Dose*time*radiation quality	9	9.47E + 09	3.60	0.0002
Residuals	457	1.33E + 11		

*Df* degree of freedom, *Sum Sq* sum of squares.

**Figure 4:**
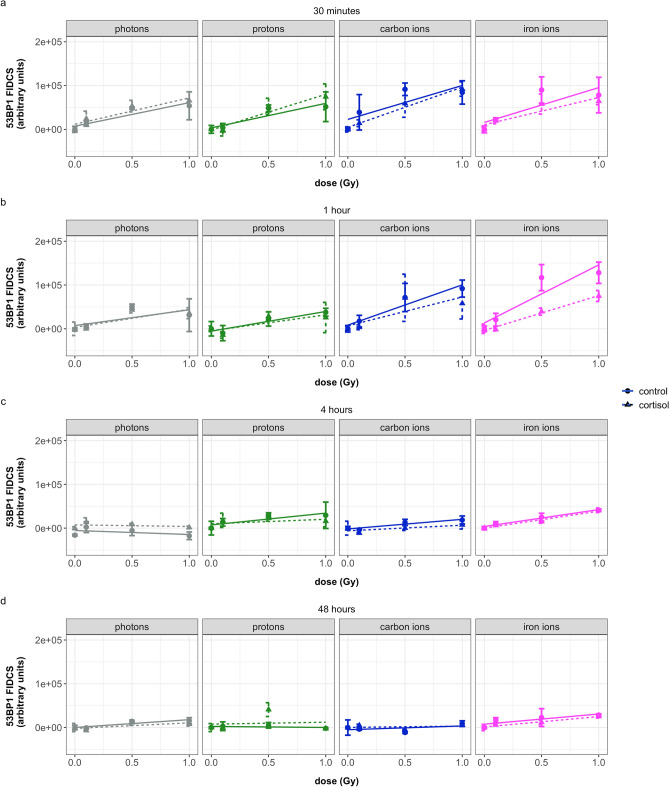
Overview of FIDCS for 53BP1 at different time points after exposure of NHDF to different radiation qualities and with or without addition of cortisol (1 µmol/L). (**a**) cell fixation at 30 min after irradiation, (**b**) cell fixation at 1 h after irradiation, (**c**) cell fixation at 4 h after irradiation, (**d**) cell fixation at 48 h after irradiation. Photons = γ-rays. FIDCS are calculated by multiplying the area by the intensity for each individual focus. Plots show the mean (dots) with standard deviations (error bars) for four replicate wells, lines show linear regression lines (dotted lines for cortisol). Values are baseline subtracted. Four wells per condition, on average 440 nuclei per well were imaged.

As observed with γ-H2AX, the FIDCS for 53BP1 increased linearly with dose. In general, the FIDCS was significantly higher after exposure to carbon and iron ions compared to photon exposed cells, this was not the case for proton exposed cells (Fig. 4).

Like γ-H2AX FIDCS, the increase in FIDCS for 53BP1 as a function of dose at one hour after iron ion exposure was significantly higher compared to other radiation qualities. At 4 and 48 h after irradiation, the slope of the FIDCS as function of dose was significantly lower compared to 30 min after exposure for all radiation qualities, although the slope for iron ion exposed cells was slightly higher compared to photons at 4 h, yet this effect was bordering significance (*p* = 0.0714).

Again, adding the overall effect of cortisol significantly improved the model. Contrary to the γ-H2AX FIDCS, where cortisol did not significantly influence the regression coefficients, the 53BP1 FIDCS was significantly lower than cells exposed to the control vehicle in carbon and iron ion exposed cells compared to photons and protons. This effect was best observed 1 h after radiation exposure (Fig. 4b).

### Effect of the radiation quality on the yield and repair of DNA damage

**Figure 5:**
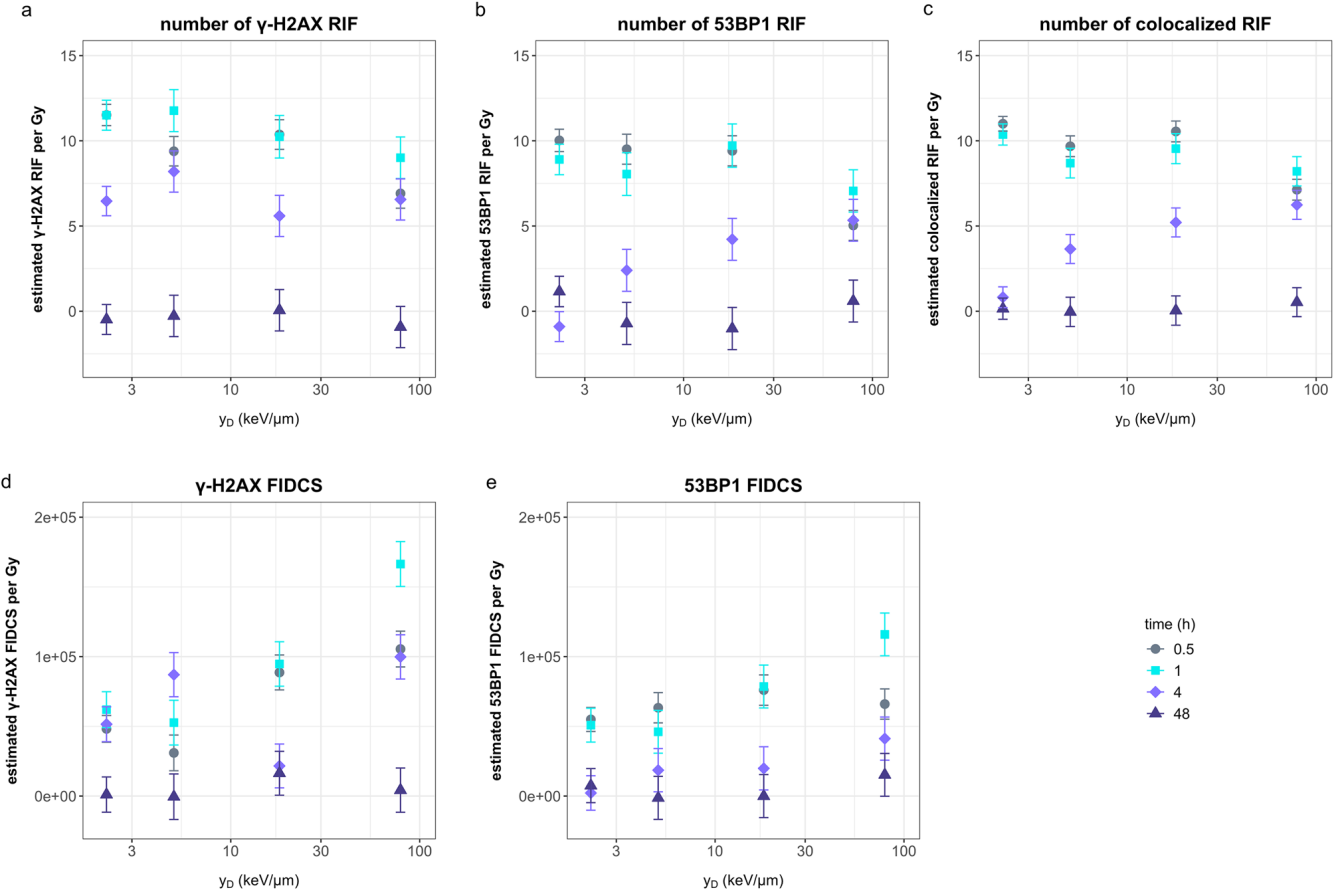
Estimated values for number of γ-H2AX RIF (**a**), 53BP1 RIF (**b**), colocalized RIF (**c**), γ-H2AX FIDCS (**d**), and 53BP1 FIDCS (**e**) as functions of the dose-mean lineal energy in water of the different radiation qualities for the different time points after irradiation. Y-values are calculated based on regression model fitted to the in vitro data as explained in paragraph 3.1 and 3.2 and Figs. 1, 3, and 4. Dots represent the slopes calculated based on the regression coefficients error bars represent the standard error of the regression coefficients. The data represents cells not exposed to cortisol.

To better understand the effects of radiation quality on DNA damage and repair over time, we used the estimated model parameters of cells not exposed to cortisol, to calculate the slope of the regression curves for the number of γ-H2AX, 53BP1, and colocalized RIF as well as the FIDCS for γ-H2AX and 53BP1. These measures were chosen since slope values provide information on the predicted amount of damage per absorbed gray. The results of these calculations were plotted as a function of the dose-mean lineal energy in water for each radiation quality (see Table 1, y_D_) and are shown in Fig. 5.

Slope values for the number of RIF for all measured foci (γ-H2AX, 53BP1, and colocalized) were lower for iron ions compared to the low lineal energy exposures during the first two time points after radiation (Fig. 5a–c). Contrary to this, slope values for FIDCS of γ-H2AX and 53BP1 showed an increase as a function of lineal energy during the first two timepoints after radiation exposure. At four hours, the largest effect of lineal energy was observed on the number of 53BP1 and colocalized RIF, with the highest amount of RIF in cells exposed to iron ions. However, for number of γ-H2AX RIF, this effect was not observed at four hours after radiation.

Slope values for γ-H2AX FIDCS increased with increase in lineal energy during the first hour after radiation. Between 30 min and 1 h after radiation a large increase in slope values was observed at high lineal energy for cells exposed to iron ions. For proton exposed cells, the peak in γ-H2AX FIDCS slope values was observed at four hours after radiation exposure. At this timepoint, the number of γ-H2AX RIF remained relatively high compared to the number of 53BP1 and colocalized RIF for proton exposed cells.

The 53BP1 FIDCS at lower lineal energy, for photon and proton exposed cells, showed reduced slope values with time. At higher lineal energy, no repair for carbon ion exposed, or increase in FIDCS for iron ion exposed cells, were observed during the first hour after radiation exposure. This was followed by a decline at 4 and 48 h. Yet, after 48 h of repair, at higher lineal energy values, the number of RIF and FIDCS slopes for γ-H2AX and 53BP1 remained slightly higher compared to values at lower lineal energy. This was best observed for number of 53BP1 and colocalized RIF and 53BP1 FIDCS for iron ions and γ-H2AX FIDCS for carbon ions.

**Figure 6:**
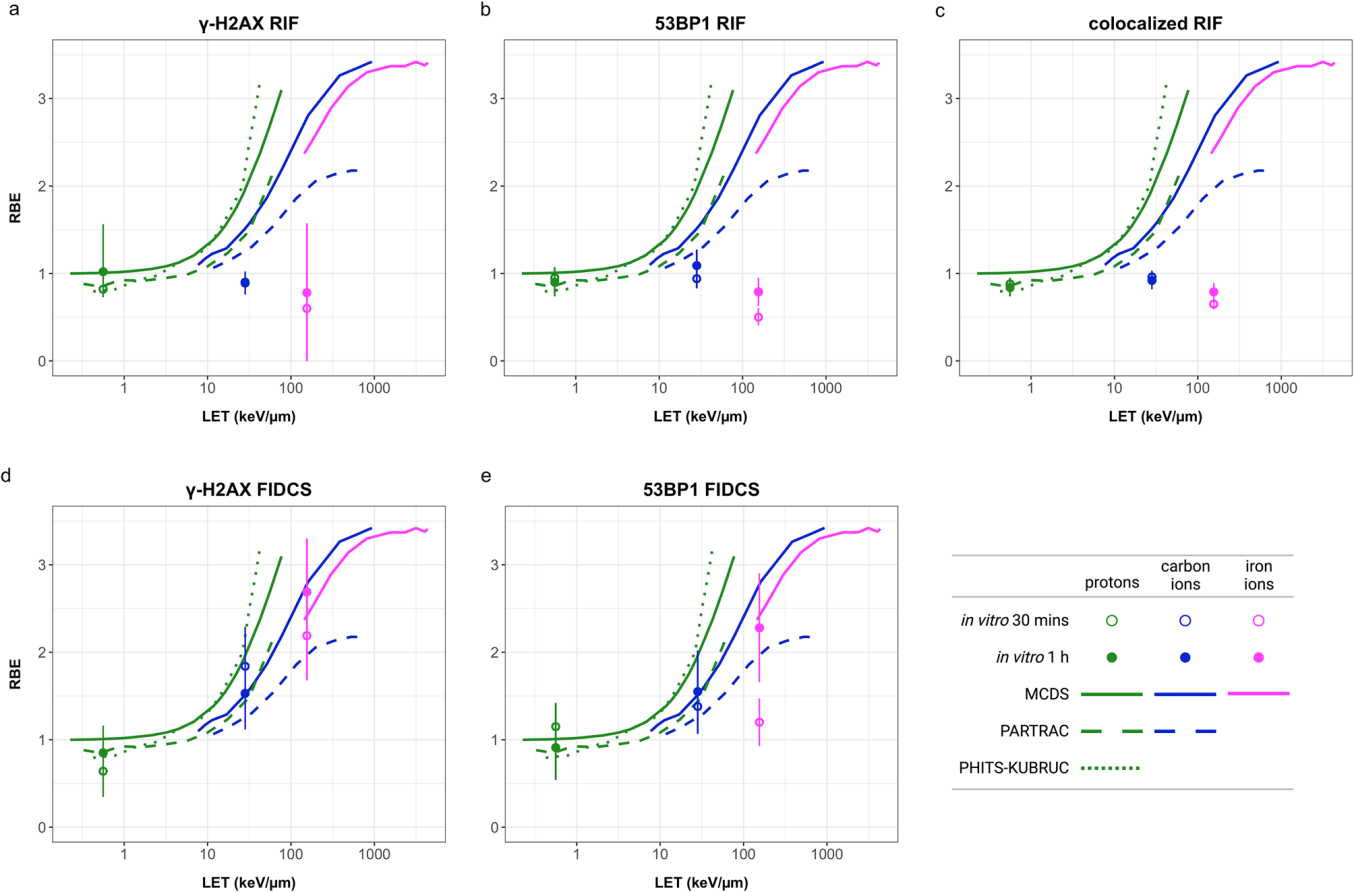
RBE values for number of γ-H2AX RIF (**a**), 53BP1 RIF (**b**), colocalized RIF (**c**), γ-H2AX FIDCS (**d**), and 53BP1 FIDCS (**e**) as functions of LET. The dots represent the average in vitro RBE as determined by dividing the slope values (estimated with the RIF or the FIDCS) for each ion by the slope values of the photon irradiated cells. The full and open symbols are the in vitro results at 30 min and 1 h after irradiation, respectively. The error bars show the mitigated uncertainties based on the standard errors of the regression coefficients. The lines represent the simulated RBE values based on novel calculations with MCDS, and published simulations with PARTRAC and PHITS*.*

Next, for the number of γ-H2AX, 53BP1, and colocalized RIF, as well as γ-H2AX FIDCS, and 53BP1 FIDCS, we divided the slope values of the initial DNA damage (30 min and 1 h) for each radiation quality by the slope value of photon exposed cells at that time point to estimate the RBE values for the initial yield of DNA DSBs. Figure 6 shows the RBE as a function of the dose-mean LET of the primary beam in water (LET_D_). For the in vitro data, this has been quantified as the RBE assessed 30 min and 1 h after irradiation, as representative for the initial DNA damage. The in vitro results were compared with corresponding novel calculations with MCDS^24^ and published simulations with PARTRAC^25^ and PHITS-KURBUC^26^.

The in vitro RBE values based on the number of RIF at 30 min and 1 h after radiation exposure in our in vitro experiment were not in agreement with the simulated initial yields of DNA DSBs at high LET. Contrary, a reasonable agreement was observed between the simulated RBE trends as a function of the LET and the corresponding experimental results using the FIDCS approach for the quantification of the radiation-induced damage.

## Discussion

In this paper, we investigated the effect of different radiation qualities on DNA double strand breaks in normal human dermal fibroblasts. Furthermore, the effect of the stress hormone cortisol on DNA strand breaks and repair was explored. Using the standard method of counting the total number of RIF based on two repair proteins (γ-H2AX and 53BP1) and their colocalization, we were not able to observe any significant differences between radiation qualities in the number of spots during the first hour after irradiation, nor did we find a significant effect of cortisol exposure on the total number of RIF. For this reason, we introduced a new metric for measuring the complexity of the RIF, the FIDCS, with which we were able to observe the effects of radiation quality and cortisol exposure. Using the FIDCS to calculate RBE values based on the slopes of the FIDCS as a function of dose at 30 min and one hour after irradiation, we found results that were in close alignment with simulation studies. In the following sections, we discuss our findings with respect to the effect of the different radiation qualities on the number of RIF and FIDCS.

With an increase in absorbed dose in the cell nucleus, an increased number of RIF, γ-H2AX, and 53BP1 FIDCS was observed, indicating increased yields of DNA DSBs during the first time points after irradiation. The initial yield of DNA DSBs per unit of dose quantified by RIF was the lowest in cells irradiated with iron ions (Fig. 1a). Contrary, FIDCS per unit of dose was the highest for cells exposed carbon and iron ions (Figs. 3 and 4a,b).

These contradictory observations may be explained based on the set-up of our experiment and the physical characteristics of the different radiation qualities. The carbon and iron ions used in our experiments had an LET of 29 and 155 keV/μm respectively. High-LET particles lose their energy more densely along their path than lower-LET photons and protons. For this reason, when these ions traverse a cell nucleus, the increased density of energy imparted leads to an increase in damage to the DNA compared to lower-LET particles^27^. In our experiments, the plane of irradiation was in the same orientation as the imaging plane, which resulted in the overlap of clustered DNA strand breaks and likely led to misidentification and an underestimation of the number of RIF, especially at exposure to carbon and iron ions^28^.

Besides, the difference in fluence between the experiments should be considered as well. Since we compared exposures at similar doses, the particle fluence changed between the different radiation qualities as a result of differences in LET. The fluence of the primary beam for iron ions at 1 Gy (4 × 10^6^ ions/cm^2^) and carbon ions (~ 2.2 × 10^7^ ions/cm^2^) was significantly lower than that of protons (~ 1.1 × 10^9^ ions/cm^2^). Because of the higher LET and lower fluence of the iron and carbon ions, energy deposition within the nuclei of the cells is more localized, resulting in clustered DNA strand breaks (two or more lesions within a few helical turns^29^). At lower LET and higher fluence, a more random pattern of RIF distribution was observed.

The results based on the newly introduced FIDCS, which is determined by the area and intensity of each focus, suggest that foci in the cells irradiated with carbon and iron ions are likely consisting of complex and clustered DNA strand breaks. This was concluded considering that the foci of cells exposed to high LET particles have greater FIDCS with respect to ones of cells exposed to low LET particles, suggesting more repair-associated proteins are present at each focus compared to photon exposed cells. These results are consistent with previous findings in which an increased number of clustered DNA strand breaks as a result of exposure to higher LET particles was reported^5,30^. Furthermore, the RBE for the initial yield of DNA DSBs quantified with the FIDCS (both γ-H2AX and 53BP1) followed the trend of simulation studies, namely higher RBE values with the increase of the LET. By contrast, the in vitro RBE quantified by RIF substantially underestimated the simulation results.

Nonetheless, one should note that, at 30 min after radiation exposure, the RBE values based on 53BP1 FIDCS were still considerably lower than the simulated data (Fig. 6). However, at 1 h after radiation, these differences were no longer observed, which could possibly be explained by a delayed response of 53BP1 to the induced damage at higher LET.

The good agreement between our in vitro RBE measurements based on the FIDCS and the simulated data is supportive of our novel proposed metric of the FIDCS, which integrates the area of the foci with the intensity of the fluorescent signal. We propose, therefore, that besides the standard quantification of DNA damage based on the number of RIF alone, FIDCS could provide an additional informative measure, especially in the case of particle irradiation when advanced microscopic techniques with increased resolution are not available.

The changes in FIDCS over time depended on radiation quality and slight differences were observed between the γ-H2AX and 53BP1 repair proteins. For cells exposed to photons and protons, the slope of 53BP1 FIDCS as a function of dose slightly decreased between the 30 min and one hour time points, indicating a reduction in the amount of 53BP1 and likely repair of the damage, which continued to lower at 4 and 48 h. 53BP1 and γ-H2AX FIDCS slope values for carbon and iron ions exposed cells remained higher at most timepoints, indicating a reduced repair of these DSBs compared to photon and proton exposed cells. For iron ions, the 53BP1 and γ-H2AX FIDCS slopes increased during the first hour, which may indicate an ongoing process of recruitment of repair proteins at the site of damage.

At 4 h after radiation exposure, the greatest differences in slope values of the number of colocalized RIF were observed between the different radiation qualities. For iron ion exposed cells, the remaining RIF at this time point was the highest compared to other groups. Interestingly, although the slope values for the number of RIF in cells exposed to protons at 4 h after irradiation were reduced, the γ-H2AX FIDCS slope at this timepoint was highest compared to previous timepoints. In a similar fashion, γ-H2AX FIDCS slopes for carbon irradiated cells at 48 h after radiation exposure were relatively high, while the total number of RIF at this timepoint was close to baseline values. This may reflect that although the number of spots may reduce with time, the spots that remain are likely more complex compared to previous time points.

These observations may be explained by considering the spatiotemporal characteristics of DNA DSBs repair. Repair of DNA damage in the highly condensed form of heterochromatin has been linked to slower repair rates. For repair complexes to be able to access the site of DNA breakage, remodeling of heterochromatin is required, including chromatin decondensation. This chromatin movement may lead to an apparent fusion of two or more foci^30–32^. Changes in chromatin structure during the DNA damage response have previously been indicated^18^ and are proposed to reflect the repair of complex lesions at later time points after radiation. The increased FIDCS in the relatively few remaining DSBs in our results might therefore reflect the repair of more complex DNA DSBs clusters.

The addition of the stress hormone cortisol to the cell culture medium did not affect the total number of RIF. However, lower FIDCS as a function of dose were observed in cortisol exposed cells. These differences were best seen 30 min and 1 h after exposure to carbon and iron ions. Previous studies have also indicated the repair of DNA damage to be affected after exposure to cortisol^16,17^. Possible mechanisms through which stress hormones can damage the DNA and influence repair are related to the induction of reactive oxygen species and modulating the transcription of genes involved in DNA damage signaling^33,34^. The reduced levels of γ-H2AX and 53BP1 FIDCS in cortisol exposed cells, may reflect a reduced capacity for recruiting repair proteins in cortisol exposed cells, particularly in cells exposed to high-LET ions. Besides, since cortisol acts as a regulator of the cell cycle^35–37^, and the phase of the cell cycle dictates which repair mechanisms for DNA DSBs are available^38^, it is plausible that cortisol-induced effects on the cell cycle may have contributed to our findings. To better understand the repair response in cortisol exposed cells, we suggest that further investigations are needed, including more time points after irradiation and at different concentrations of cortisol to better understand the spatiotemporal characteristics of the DNA damage response in cortisol exposed cells. In addition, molecular studies aiming to elucidate the mechanisms underlying these observations, as well as information on cell cycle phase, could aid in improving our understanding of the interactions between stress hormones, ionizing radiation, and the repair of DNA DSBs. Finally, although the FIDCS in our study is aimed to be an affordable way of measuring DNA damage, it would be of interest to acquire a dataset of high-resolution DNA DSBs in cortisol exposed irradiated cells to better understand the effect of cortisol on the DNA DSB repair at the nanoscale.

In conclusion, using a new metric of FIDCS for quantification of DNA damage after exposure to ionizing radiation, we were able to indicate an increase in DNA damage complexity after exposure to high-LET carbon and iron ions compared to photons and protons. Besides, using this FIDCS, we found differences in DNA damage response between cortisol exposed and control cells. This indicates that exposure to ionizing radiation in combination with cortisol may reduce the recruitment of proteins and the lesions site, although more research is needed to better understand this observed effect. The results of this study highlight the potential of our newly introduced FIDCS for assessing DNA damage, especially after exposure to ions at high-LET. The conventional quantification method for DNA damage, i.e. counting the number of RIF, fails to account for the heterogeneity and complexity of each individual DNA damage focus. By considering the intensity and area of each focus, the FIDCS allows for a more nuanced evaluation of the complexity of DNA damage induced by heavy ions. Therefore, the FIDCS may provide additional valuable information regarding the severity and repair potential of each lesion.

### Supplementary Information

Supplementary Information.

## Data Availability

**T**he datasets used and/or analyzed during the current study available from the corresponding author on reasonable request.
