# Supplementary material for: Radiation-induced DNA double-strand breaks in cortisol exposed fibroblasts as quantified with the novel foci-integrated damage complexity score (FIDCS)
Source: Sci Rep. 2024 May 6;14:10400. doi: 10.1038/s41598-024-60912-y (PMC11074260; doi:10.1038/s41598-024-60912-y)
Supplement: Supplementary file 1 — Supplementary Information. [file 41598_2024_60912_MOESM1_ESM.docx]

**SUPPLEMENTARY MATERIALS TO**

**Space simulated radiation-induced DNA double-strand breaks in cortisol exposed fibroblast as quantified with the novel Foci-Integrated Damage Complexity Score (FIDCS)**

**Wilhelmina E. Radstake ^1, 2^, Alessio Parisi ^3,4^, Silvana Miranda ^1, 2^, Kiran Gautam ^1^, Randy Vermeesen ^1^, Emil Rehnberg ^1, 2^, Kevin Tabury ^1,5^, Rob Coppes ^6,7^, Marc-Jan van Goethem ^7^, Sytze Brandenburg ^7^, Ulrich Weber ^8^, Claudia Fournier ^8^, Marco Durante ^8,9^, Bjorn Baselet ^1*^, and Sarah Baatout ^1,2^**

**SM1. Radiation transport computer simulations**

Radiation transport simulations were performed using the Particle and Heavy Ion Transport code System (PHITS, Sato et al 2018) version 3.28. A simplified geometry of the cell holder and of the material between the radiation source (i.e. the exit of the nozzle) and the cell holders (i.e. build up slabs) was implemented in PHITS. The simulated holder was filled with liquid water representing the cells and the medium. The simulated holder was irradiated with monoenergetic beams (energy listed in **Table 1** of the manuscript) impinging perpendicularly its front surface.

The following quantities were assessed in the first 100 µm of water within the holder (as representative of the position of the attached cells): dose-mean unrestricted linear energy transfer (LET) in water of the primary beam, dose-mean unrestricted LET in water of all particles (primary beam and all secondary particles), dose-mean lineal energy within homogenously distributed liquid water spheres with diameter equal to 0.6 µm. This dimension was chosen as representative of the scale where the accumulation and repair of DNA damage correlates with cell death (Parisi et al., 2022).

The following physical models and options were used in the PHITS simulations:

- Macroscopic energy loss:
  - Charged particles (except of electrons and positrons): ATIMA.
  - Photons, electrons, and positrons: EGS5.
- Transport cutoffs: 1 keV/n (ions), 1 keV (all other particles but neutrons), 10^-8^ keV (neutrons).
- Angular straggling: Lynch’s Coulomb diffusion formula based on Moliere theory.
- Energy straggling: Landau-Vavilov formula.
- Transport and interaction of low energy neutrons: event generator mode v2.
- Mean ionization potential of water = 78 eV.
- Nuclear reactions: default PHITS models.
- Microdosimetry: PHITS microdosimetric function.

More details can be found in Sato et al 2018, the PHITS manual (<https://phits.jaea.go.jp/manual/manualE-phits.pdf>), and references therein.

**SM2. Tables of regression model outputs**

Table S1. Estimates for the regression model for the number of colocalized RIF.

| variable | coefficients | standard error | t-value | p-value |
| --- | --- | --- | --- | --- |
| nuclear area | 0.002 | 0.001 | 1.85 | 0.0650 |
| xcort | -0.21 | 0.09 | -2.37 | 0.0181 |
| xt1 | 0.86 | 0.33 | 2.60 | 0.0097 |
| xt4 | 1.31 | 0.34 | 3.86 | 0.0001 |
| xt48 | 0.44 | 0.34 | 1.32 | 0.1887 |
| xd | 11.00 | 0.44 | 25.26 | < 0.0001 |
| xprot | 0.22 | 0.33 | 0.65 | 0.5162 |
| xC | 0.45 | 0.34 | 1.32 | 0.1866 |
| xFe | 0.98 | 0.33 | 2.93 | 0.0036 |
| xt1 * xd | -0.63 | 0.61 | -1.02 | 0.3077 |
| xt4 * xd | -10.18 | 0.60 | -16.90 | < 0.0001 |
| xt48 * xd | -10.84 | 0.61 | -17.67 | < 0.0001 |
| xt1 * xprot | -1.00 | 0.47 | -2.12 | 0.0346 |
| xt4 * xprot | -0.28 | 0.47 | -0.60 | 0.5470 |
| xt48 * xprot | -0.16 | 0.47 | -0.34 | 0.7306 |
| xt1 * xC | -0.89 | 0.47 | -1.89 | 0.0594 |
| xt4 * xC | -1.32 | 0.47 | -2.80 | 0.0054 |
| xt48 * xC | -0.44 | 0.47 | -0.94 | 0.3486 |
| xt1 * xFe | -1.20 | 0.47 | -2.55 | 0.0110 |
| xt4 * xFe | -1.71 | 0.47 | -3.63 | 0.0003 |
| xt48 * xFe | -0.93 | 0.47 | -1.97 | 0.0493 |
| xd * xprot | -1.32 | 0.60 | -2.18 | 0.0297 |
| xd * xC | -0.45 | 0.61 | -0.74 | 0.4625 |
| xd * xFe | -3.87 | 0.60 | -6.42 | < 0.0001 |
| xt1 * xd * xprot | -0.37 | 0.86 | -0.43 | 0.6697 |
| xt4 * xd * xprot | 4.15 | 0.84 | 4.91 | < 0.0001 |
| xt48 * xd * xprot | 1.12 | 0.85 | 1.32 | 0.1878 |
| xt1 * xd * xC | -0.38 | 0.87 | -0.44 | 0.6591 |
| xt4 * xd * xC | 5.06 | 0.84 | 6.00 | < 0.0001 |
| xt48 * xd * xC | 0.32 | 0.85 | 0.37 | 0.7090 |
| xt1 * xd * xFe | 1.71 | 0.85 | 2.01 | 0.0452 |
| xt4 * xd * xFe | 9.30 | 0.84 | 11.01 | < 0.0001 |
| xt48 * xd * xFe | 4.25 | 0.84 | 5.04 | < 0.0001 |

Table S2. ANOVA table for the regression model of average number of γ-H2AX RIF.

|  | Df | Sum Sq | F value | p-value |
| --- | --- | --- | --- | --- |
| nuclear area | 1 | 0.4 | 0.24 | 0.6269 |
| time | 3 | 1238.1 | 233.05 | < 0.0001 |
| stress | 1 | 3.3 | 1.87 | 0.1724 |
| radiation quality | 3 | 48.4 | 9.12 | < 0.0001 |
| dose | 1 | 3494.6 | 1973.29 | < 0.0001 |
| time*stress | 3 | 1 | 0.20 | 0.8980 |
| time*radiation quality | 9 | 60 | 3.77 | 0.0001 |
| stress*radiation quality | 3 | 8.3 | 1.56 | 0.1979 |
| time*dose | 3 | 1361.3 | 256.23 | < 0.0001 |
| ion*dose | 3 | 44.7 | 8.41 | < 0.0001 |
| time*stress*radiation quality | 9 | 48.2 | 3.02 | 0.0016 |
| time*radiation quality*dose | 9 | 53.9 | 3.38 | 0.0005 |
| residuals | 456 | 807.6 |  |  |

The model was fitted with two three-way interactions between time, stress, and radiation quality and time, dose, and radiation quality. In addition, the nuclear area was added to the model as covariate. Df = degree of freedom, Sum Sq = sum of squares

Table S3. Estimates for the regression model for the average number of γ-H2AX RIF.

| variable | coefficients | standard error | t-value | p-value |
| --- | --- | --- | --- | --- |
| nuclear area | 0.01 | 0.00 | 5.45 | <0.0001 |
| x_d_ | 11.52 | 0.62 | 18.46 | <0.0001 |
| x_cort_ | -0.32 | 0.48 | -0.67 | 0.5042 |
| x_t1_ | 1.15 | 0.58 | 1.98 | 0.0482 |
| x_t4_ | 2.43 | 0.59 | 4.09 | 0.0001 |
| x_t48_ | 1.59 | 0.59 | 2.71 | 0.0070 |
| x_prot_ | 0.81 | 0.58 | 1.39 | 0.1663 |
| x_C_ | 0.39 | 0.59 | 0.66 | 0.5098 |
| x_Fe_ | 1.52 | 0.58 | 2.62 | 0.0091 |
| x_t1_ * x_cort_ | 0.33 | 0.68 | 0.49 | 0.6225 |
| x_t4_ * x_cort_ | 1.22 | 0.67 | 1.81 | 0.0711 |
| x_t48_ * x_cort_ | -0.60 | 0.67 | -0.89 | 0.3726 |
| x_t1_ * x_prot_ | -2.99 | 0.82 | -3.63 | 0.0003 |
| x_t4_ * x_prot_ | -2.00 | 0.82 | -2.43 | 0.0155 |
| x_t48_ * x_prot_ | -2.38 | 0.83 | -2.88 | 0.0041 |
| x_t1_ * x_C_ | -1.38 | 0.82 | -1.68 | 0.0942 |
| x_t4_ * x_C_ | -1.02 | 0.82 | -1.24 | 0.2173 |
| x_t48_ * x_C_ | -0.73 | 0.83 | -0.88 | 0.3799 |
| x_t1_ * x_Fe_ | -0.85 | 0.82 | -1.03 | 0.3026 |
| x_t4_ * x_Fe_ | -1.91 | 0.82 | -2.32 | 0.0207 |
| x_t48_ * x_Fe_ | -2.13 | 0.82 | -2.59 | 0.0100 |
| x_prot_* x_cort_ | -0.89 | 0.67 | -1.32 | 0.1875 |
| x_prot_* x_C_ | 0.58 | 0.67 | 0.86 | 0.3906 |
| x_prot_* x_Fe_ | 0.68 | 0.67 | 1.02 | 0.3103 |
| x_t1_ * x_d_ | -0.01 | 0.88 | -0.02 | 0.9879 |
| x_t4_ * x_d_ | -5.05 | 0.86 | -5.86 | <0.0001 |
| x_t48_ * x_d_ | -12.00 | 0.88 | -13.65 | <0.0001 |
| x_d_ * x_prot_ | -2.13 | 0.86 | -2.46 | 0.0142 |
| x_d_ * x_C_ | -1.15 | 0.87 | -1.32 | 0.1876 |
| x_d_ * x_Fe_ | -4.61 | 0.86 | -5.33 | 0.0000 |
| x_t1_ * x_cort_ * x_prot_ | 1.72 | 0.95 | 1.80 | 0.0727 |
| x_t4_ * x_cort_ * x_prot_ | -0.57 | 0.95 | -0.60 | 0.5460 |
| x_t48_ * x_cort_ * x_prot_ | 2.72 | 0.95 | 2.88 | 0.0042 |
| x_t1_ * x_cort_ * x_C_ | -1.06 | 0.97 | -1.10 | 0.2724 |
| x_t4_ * x_cort_ * x_C_ | -1.74 | 0.95 | -1.84 | 0.0666 |
| x_t48_ * x_cort_ * x_C_ | 0.25 | 0.95 | 0.26 | 0.7925 |
| x_t1_ * x_cort_ * x_Fe_ | -1.29 | 0.95 | -1.36 | 0.1758 |
| x_t4_ * x_cort_ * x_Fe_ | -2.44 | 0.95 | -2.58 | 0.0101 |
| x_t48_ * x_cort_ * x_Fe_ | 0.10 | 0.95 | 0.11 | 0.9166 |
| x_t1_ * x_d_ * x_prot_ | 2.40 | 1.23 | 1.95 | 0.0524 |
| x_t4_ * x_d_ * x_prot_ | 3.86 | 1.21 | 3.19 | 0.0015 |
| x_t48_ * x_d_ * x_prot_ | 2.34 | 1.22 | 1.92 | 0.0552 |
| x_t1_ * x_d_ * x_C_ | -0.12 | 1.25 | -0.09 | 0.9265 |
| x_t4_ * x_d_ * x_C_ | 0.28 | 1.21 | 0.23 | 0.8188 |
| x_t48_ * x_d_ * x_C_ | 1.69 | 1.22 | 1.39 | 0.1651 |
| x_t1_ * x_d_ * x_Fe_ | 2.11 | 1.22 | 1.73 | 0.0845 |
| x_t4_ * x_d_ * x_Fe_ | 4.70 | 1.21 | 3.89 | 0.0001 |
| x_t48_ * x_d_ * x_Fe_ | 4.16 | 1.21 | 3.45 | 0.0006 |

Table S4. ANOVA table for the regression model of average number of 53BP1 RIF.

|  | Df | Sum Sq | F value | p-value |
| --- | --- | --- | --- | --- |
| nuclear area | 1 | 114.55 | 62.36 | < 0.0001 |
| dose | 1 | 1924.15 | 1047.56 | < 0.0001 |
| stress | 1 | 0.75 | 0.41 | 0.5219 |
| time | 3 | 788.86 | 143.16 | < 0.0001 |
| radiation quality | 3 | 5.89 | 1.07 | 0.3618 |
| dose*stress | 1 | 12.70 | 6.92 | 0.0088 |
| dose*time | 3 | 1028.54 | 186.66 | < 0.0001 |
| time*radiation quality | 9 | 185.80 | 11.24 | < 0.0001 |
| dose*radiation quality | 3 | 11.13 | 2.02 | 0.1103 |
| dose*time* radiation quality | 9 | 210.85 | 12.75 | < 0.0001 |
| residuals | 470 | 863.29 |  |  |

The model was fitted with a two-way interaction between dose and stress, and a three-way interaction between dose, time, and radiation quality. Nuclear area was added as covariate. Df = degree of freedom, Sum Sq = sum of squares

Table S5. Estimates for the regression model for the number of 53BP1 RIF.

| variable | coefficients | standard error | t-value | p-value |
| --- | --- | --- | --- | --- |
| nuclear area | 0.00 | 0.00 | 1.69 | 0.0910 |
| x_d_ | 10.02 | 0.66 | 15.27 | < 0.0001 |
| x_cort_ | -0.21 | 0.17 | -1.22 | 0.2215 |
| x_t1_ | 1.26 | 0.49 | 2.59 | 0.0100 |
| x_t4_ | 2.05 | 0.49 | 4.14 | < 0.0001 |
| x_t48_ | 0.58 | 0.49 | 1.18 | 0.2372 |
| x_prot_ | -0.39 | 0.49 | -0.80 | 0.4250 |
| x_C_ | 0.49 | 0.49 | 1.00 | 0.3177 |
| x_Fe_ | 1.31 | 0.49 | 2.70 | 0.0073 |
| x_d_ * x_cort_ | 0.59 | 0.31 | 1.92 | 0.0558 |
| x_t1_ * x_d_ | -1.12 | 0.90 | -1.25 | 0.2123 |
| x_t4_ * x_d_ | -10.93 | 0.88 | -12.44 | < 0.0001 |
| x_t48_ * x_d_ | -8.87 | 0.89 | -9.92 | < 0.0001 |
| x_t1_ * x_prot_ | -1.09 | 0.69 | -1.59 | 0.1127 |
| x_t4_ * x_prot_ | 1.00 | 0.69 | 1.46 | 0.1458 |
| x_t48_ * x_prot_ | 0.67 | 0.69 | 0.96 | 0.3365 |
| x_t1_ * x_C_ | -1.23 | 0.69 | -1.79 | 0.0745 |
| x_t4_ * x_C_ | -2.34 | 0.69 | -3.40 | 0.0007 |
| x_t48_ * x_C_ | -0.72 | 0.69 | -1.05 | 0.2955 |
| x_t1_ * x_Fe_ | -2.11 | 0.68 | -3.09 | 0.0021 |
| x_t4_ * x_Fe_ | -2.62 | 0.68 | -3.83 | 0.0001 |
| x_t48_ * x_Fe_ | -0.87 | 0.69 | -1.27 | 0.2042 |
| x_d_ * x_prot_ | -0.52 | 0.88 | -0.59 | 0.5565 |
| x_d_ * x_C_ | -0.61 | 0.88 | -0.69 | 0.4887 |
| x_d_ * x_Fe_ | -4.99 | 0.88 | -5.67 | < 0.0001 |
| x_t1_ * x_d_ * x_prot_ | -0.34 | 1.25 | -0.27 | 0.7851 |
| x_t4_ * x_d_ * x_prot_ | 3.82 | 1.23 | 3.10 | 0.0020 |
| x_t48_ * x_d_ * x_prot_ | -1.36 | 1.24 | -1.10 | 0.2734 |
| x_t1_ * x_d_ * x_C_ | 1.43 | 1.27 | 1.12 | 0.2625 |
| x_t4_ * x_d_ * x_C_ | 5.73 | 1.23 | 4.66 | < 0.0001 |
| x_t48_ * x_d_ * x_C_ | -1.56 | 1.24 | -1.26 | 0.2079 |
| x_t1_ * x_d_ * x_Fe_ | 3.14 | 1.24 | 2.53 | 0.0117 |
| x_t4_ * x_d_ * x_Fe_ | 11.23 | 1.23 | 9.13 | < 0.0001 |
| x_t48_ * x_d_ * x_Fe_ | 4.43 | 1.23 | 3.60 | 0.0003 |

Table S6. Estimates for the regression model for the FIDCS of γ-H2AX.

| variable | coefficients | standard error | t-value | p-value |
| --- | --- | --- | --- | --- |
| nuclear area | 82.77 | 21.28 | 3.89 | 0.0001 |
| x_d_ | 48162.77 | 9614.99 | 5.01 | <0.0001 |
| x_cort_ | -1639.38 | 7773.86 | -0.21 | 0.8331 |
| x_t1_ | 3216.56 | 8089.08 | 0.40 | 0.6911 |
| x_t4_ | 19277.64 | 8199.15 | 2.35 | 0.0192 |
| x_t48_ | 16271.65 | 8157.21 | 2.00 | 0.0467 |
| x_prot_ | 7729.92 | 8096.81 | 0.96 | 0.3403 |
| x_C_ | 3136.35 | 8155.42 | 0.39 | 0.7007 |
| x_Fe_ | 18565.01 | 8091.57 | 2.29 | 0.0222 |
| x_d_ * x_cort_ | -8458.97 | 10588.28 | -0.80 | 0.4248 |
| x_t1_ * x_d_ | 13773.31 | 12859.71 | 1.07 | 0.2847 |
| x_t4_ * x_d_ | 3345.80 | 12499.88 | 0.27 | 0.7891 |
| x_t48_ * x_d_ | -47131.38 | 12626.12 | -3.73 | 0.0002 |
| x_t1_ * x_cort_ | 9282.83 | 10060.58 | 0.92 | 0.3567 |
| x_t4_ * x_cort_ | 9894.72 | 9929.32 | 1.00 | 0.3195 |
| x_t48_ * x_cort_ | -8324.45 | 9947.29 | -0.84 | 0.4031 |
| x_d_ * x_prot_ | -17233.84 | 12832.14 | -1.34 | 0.1800 |
| x_d_ * x_C_ | 40463.37 | 12532.80 | 3.23 | 0.0013 |
| x_d_ * x_Fe_ | 57249.95 | 12836.89 | 4.46 | <0.0001 |
| x_cort_ * x_C_ | -4989.96 | 9964.73 | -0.50 | 0.6168 |
| x_cort_ * x_Fe_ | 2433.55 | 9914.48 | 0.25 | 0.8062 |
| x_cort_ * x_C_ | -8687.89 | 10039.81 | -0.87 | 0.3873 |
| x_t1_ * x_prot_ | -16861.88 | 10821.71 | -1.56 | 0.1199 |
| x_t4_ * x_prot_ | -9206.78 | 10841.84 | -0.85 | 0.3962 |
| x_t48_ * x_prot_ | -15420.43 | 10972.34 | -1.41 | 0.1606 |
| x_t1_ * x_C_ | -2581.59 | 10819.09 | -0.24 | 0.8115 |
| x_t4_ * x_C_ | -3910.81 | 10968.99 | -0.36 | 0.7216 |
| x_t48_ * x_C_ | -9425.33 | 10853.76 | -0.87 | 0.3857 |
| x_t1_ * x_Fe_ | -1497.62 | 10823.56 | -0.14 | 0.8900 |
| x_t4_ * x_Fe_ | -36954.84 | 10866.78 | -3.40 | 0.0007 |
| x_t48_ * x_Fe_ | -27184.58 | 10871.69 | -2.50 | 0.0128 |
| x_t1_ * x_d_ * x_cort_ | -17586.01 | 11253.03 | -1.56 | 0.1188 |
| x_t4_ * x_d_ * x_cort_ | 6495.46 | 11201.74 | 0.58 | 0.5623 |
| x_t48_ * x_d_ * x_cort_ | 12925.04 | 11318.58 | 1.14 | 0.2541 |
| x_d_ * x_cort_ * x_prot_ | 7364.72 | 11417.90 | 0.65 | 0.5193 |
| x_d_ * x_cort_ * x_C_ | -16976.33 | 11121.89 | -1.53 | 0.1276 |
| x_d_ * x_cort_ * x_Fe_ | -14752.43 | 11172.70 | -1.32 | 0.1874 |
| x_t1_ * x_cort_ * x_prot_ | 5132.42 | 12497.20 | 0.41 | 0.6815 |
| x_t4_ * x_cort_ * x_prot_ | -20914.29 | 12379.51 | -1.69 | 0.0918 |
| x_t48_ * x_cort_ * x_prot_ | 12218.39 | 12592.00 | 0.97 | 0.3324 |
| x_t1_ * x_cort_ * x_C_ | -15765.85 | 12440.84 | -1.27 | 0.2057 |
| x_t4_ * x_cort_ * x_C_ | -18800.95 | 12367.21 | -1.52 | 0.1292 |
| x_t48_ * x_cort_ * x_C_ | 12055.23 | 12324.36 | 0.98 | 0.3285 |
| x_t1_ * x_cort_ * x_Fe_ | -18527.13 | 12539.37 | -1.48 | 0.1402 |
| x_t4_ * x_cort_ * x_Fe_ | 6304.10 | 12528.31 | 0.50 | 0.6151 |
| x_t48_ * x_cort_ * x_Fe_ | 22135.51 | 12477.04 | 1.77 | 0.0767 |
| x_t1_ * x_d_ * x_prot_ | 7933.76 | 16065.89 | 0.49 | 0.6217 |
| x_t4_ * x_d_ * x_prot_ | 52766.37 | 15831.78 | 3.33 | 0.0009 |
| x_t48_ * x_d_ * x_prot_ | 15710.48 | 16325.49 | 0.96 | 0.3364 |
| x_t1_ * x_d_ * x_C_ | -7692.03 | 15925.86 | -0.48 | 0.6293 |
| x_t4_ * x_d_ * x_C_ | -70384.17 | 15720.24 | -4.48 | <0.0001 |
| x_t48_ * x_d_ * x_C_ | -25154.79 | 15725.32 | -1.60 | 0.1104 |
| x_t1_ * x_d_ * x_Fe_ | 47210.69 | 16095.95 | 2.93 | 0.0035 |
| x_t4_ * x_d_ * x_Fe_ | -8968.90 | 15867.30 | -0.57 | 0.5722 |
| x_t48_ * x_d_ * x_Fe_ | -54059.02 | 15873.69 | -3.41 | 0.0007 |

Table S7. Estimates for the regression model for the FIDCS of 53BP1

| variable | coefficients | standard error | t-value | p-value |
| --- | --- | --- | --- | --- |
| nuclear area | 122.56 | 20.03 | 6.118 | <0.0001 |
| x_d_ | 54922.13 | 8581.96 | 6.4 | <0.0001 |
| x_cort_ | 1801.84 | 6787.79 | 0.265 | 0.7908 |
| x_t1_ | -4741.8 | 7749.39 | -0.612 | 0.5409 |
| x_t4_ | -7871.27 | 8268.94 | -0.952 | 0.3416 |
| x_t48_ | -141.75 | 7815.01 | -0.018 | 0.9855 |
| x_prot_ | -3150.39 | 7473.01 | -0.422 | 0.6735 |
| x_C_ | 20290.89 | 7511.17 | 2.701 | 0.0072 |
| x_Fe_ | 15847.19 | 7449.9 | 2.127 | 0.0339 |
| x_d_ * x_cort_ | 8802.28 | 7677.3 | 1.147 | 0.2522 |
| x_t1_ * x_d_ | -4108.31 | 12131.39 | -0.339 | 0.7350 |
| x_t4_ * x_d_ | -52743.67 | 12382.16 | -4.26 | <0.0001 |
| x_t48_ * x_d_ | -47455.18 | 12236.62 | -3.878 | 0.0001 |
| x_t1_ * x_cort_ | 4768.95 | 9593.13 | 0.497 | 0.6193 |
| x_t4_ * x_cort_ | 11419.93 | 9804.58 | 1.165 | 0.2447 |
| x_t48_ * x_cort_ | -7818.09 | 9583.07 | -0.816 | 0.4150 |
| x_cort_ * x_C_ | -4512.4 | 8556.25 | -0.527 | 0.5982 |
| x_cort_ * x_Fe_ | -20693.85 | 8546.13 | -2.421 | 0.0158 |
| x_cort_ * x_C_ | -17384.2 | 8549.38 | -2.033 | 0.0426 |
| x_t1_ * x_prot_ | -4807.6 | 10527.29 | -0.457 | 0.6481 |
| x_t4_ * x_prot_ | 19933.82 | 10831.28 | 1.84 | 0.0664 |
| x_t48_ * x_prot_ | -117.11 | 10593.86 | -0.011 | 0.9912 |
| x_t1_ * x_C_ | -9027.11 | 10527.92 | -0.857 | 0.3916 |
| x_t4_ * x_C_ | -11922.71 | 10827.83 | -1.101 | 0.2714 |
| x_t48_ * x_C_ | -25510.82 | 10574.87 | -2.412 | 0.0162 |
| x_t1_ * x_Fe_ | 3209.77 | 10521.67 | 0.305 | 0.7605 |
| x_t4_ * x_Fe_ | -5641.91 | 10808.32 | -0.522 | 0.6019 |
| x_t48_ * x_Fe_ | -11802.23 | 10550.12 | -1.119 | 0.2639 |
| x_d_ * x_prot_ | 8379.87 | 10863.79 | 0.771 | 0.4409 |
| x_d_ * x_C_ | 21064.6 | 10904.61 | 1.932 | 0.0540 |
| x_d_ * x_Fe_ | 11045.38 | 10860.45 | 1.017 | 0.3097 |
| x_t1_ * x_d_ * x_cort_ | -31483.47 | 10867.37 | -2.897 | 0.0039 |
| x_t4_ * x_d_ * x_cort_ | -16494.01 | 10914.55 | -1.511 | 0.1314 |
| x_t48_ * x_d_ * x_cort_ | -10444.09 | 10857.65 | -0.962 | 0.3366 |
| x_t1_ * x_cort_ * x_prot_ | -2205.31 | 12094.56 | -0.182 | 0.8554 |
| x_t4_ * x_cort_ * x_prot_ | -15692.28 | 12202.71 | -1.286 | 0.1991 |
| x_t48_ * x_cort_ * x_prot_ | 14900.55 | 12084.01 | 1.233 | 0.2182 |
| x_t1_ * x_cort_ * x_C_ | 5756.92 | 12096.88 | 0.476 | 0.6344 |
| x_t4_ * x_cort_ * x_C_ | -3308.9 | 12198.46 | -0.271 | 0.7863 |
| x_t48_ * x_cort_ * x_C_ | 29537.37 | 12092.05 | 2.443 | 0.0150 |
| x_t1_ * x_cort_ * x_Fe_ | -17560.2 | 12083.04 | -1.453 | 0.1468 |
| x_t4_ * x_cort_ * x_Fe_ | 1503.26 | 12199.94 | 0.123 | 0.9020 |
| x_t48_ * x_cort_ * x_Fe_ | 19456.48 | 12087.77 | 1.61 | 0.1082 |
| x_t1_ * x_d_ * x_prot_ | -13113.78 | 15355.37 | -0.854 | 0.3935 |
| x_t4_ * x_d_ * x_prot_ | 7974.86 | 15484.98 | 0.515 | 0.6068 |
| x_t48_ * x_d_ * x_prot_ | -17178.99 | 15410.93 | -1.115 | 0.2656 |
| x_t1_ * x_d_ * x_C_ | 6663.65 | 15374.36 | 0.433 | 0.6649 |
| x_t4_ * x_d_ * x_C_ | -3407.82 | 15464 | -0.22 | 0.8257 |
| x_t48_ * x_d_ * x_C_ | -28589.39 | 15409.48 | -1.855 | 0.0642 |
| x_t1_ * x_d_ * x_Fe_ | 54051.52 | 15345.28 | 3.522 | 0.0005 |
| x_t4_ * x_d_ * x_Fe_ | 27958.48 | 15470.73 | 1.807 | 0.0714 |
| x_t48_ * x_d_ * x_Fe_ | -3319.81 | 15349.86 | -0.216 | 0.8289 |

**SM3. Figures**

**
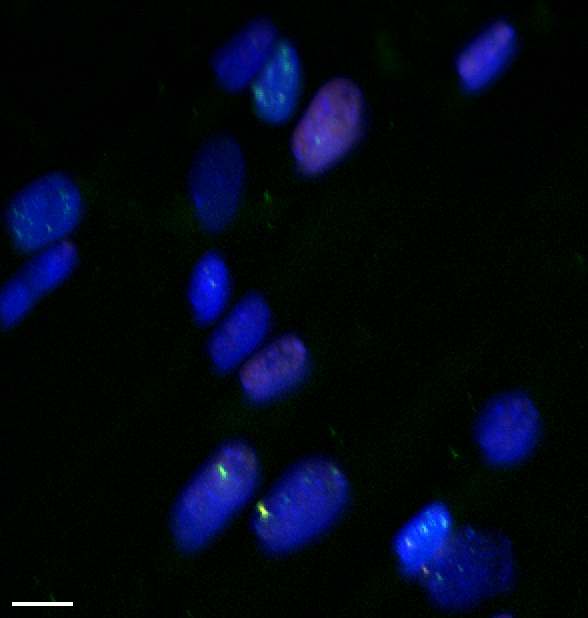
**

Figure S1. Example of unirradiated cells fixed at 30 minutes after sham-irradiation. γ-H2AX (green), and 53BP1 (orange) and DAPI for cell nuclei (blue). Scalebar = 20µm.


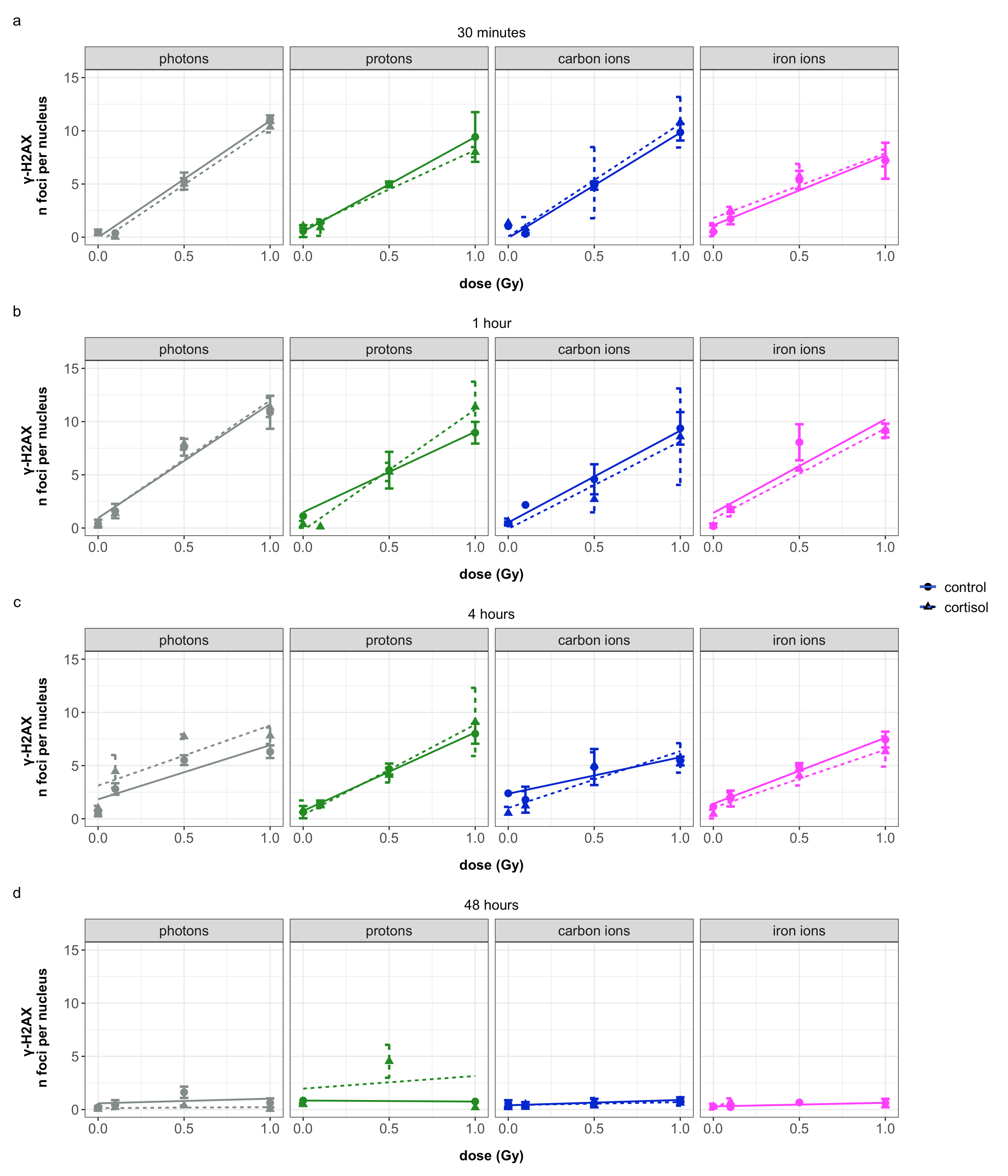
Figure S2 Overview of number of γ-H2AX foci at different time points after exposure of NHDF to different radiation qualities, and with or without addition of cortisol (1 µM). a: cell fixation at 30 minutes after irradiation, b: cell fixation at 1 hour after irradiation, c: cell fixation at 4 hours after irradiation, d: cell fixation at 48 hours after irradiation. Photons = γ-rays. Plots show mean (dots) with standard deviations (error bars), lines show linear regression lines. Values are baseline subtracted. Four wells per condition, on average 440 nuclei per well were imaged.


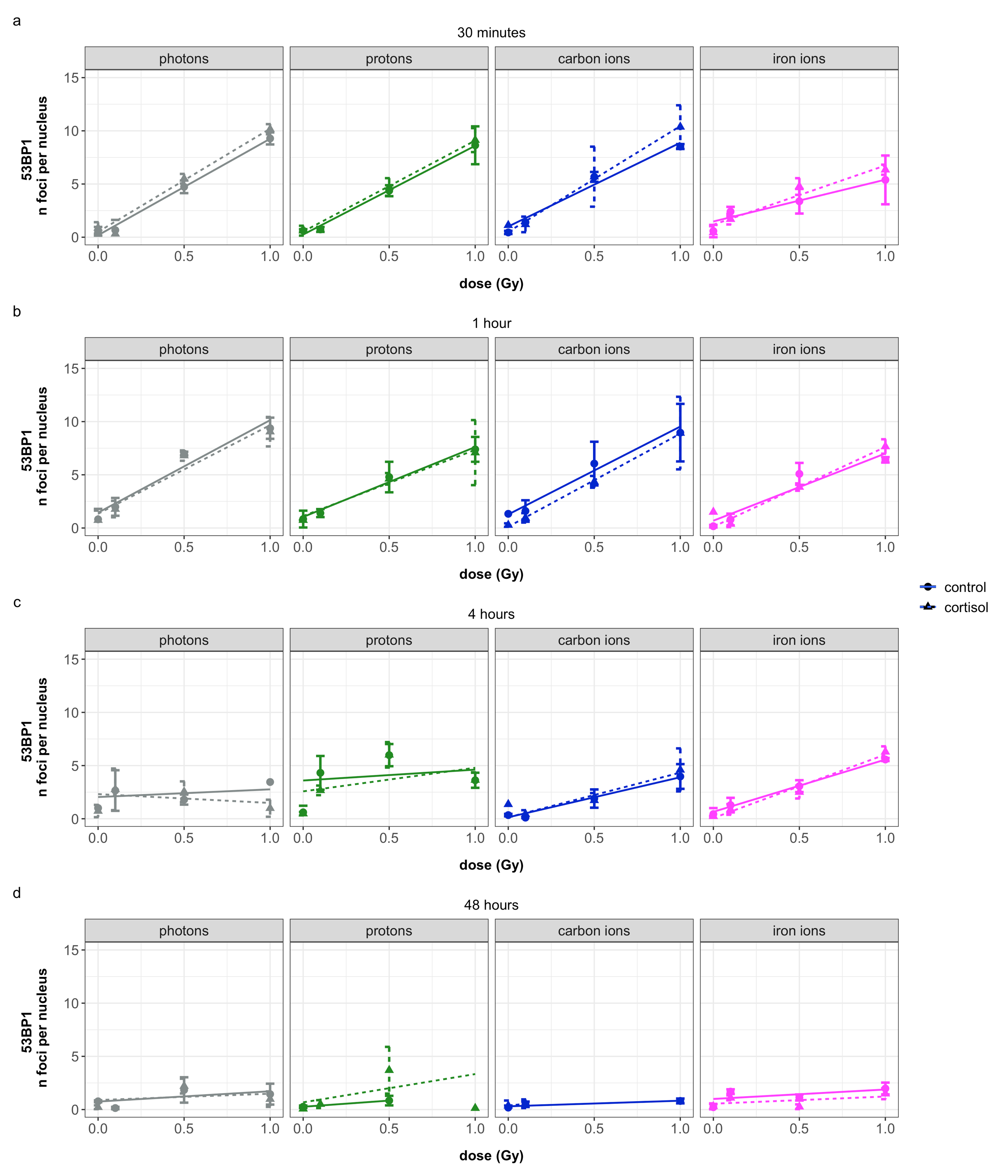


Figure S3. Overview of number of 53BP1 foci at different time points after exposure of NHDF to different radiation qualities, and with or without addition of cortisol (1 µM). a: cell fixation at 30 minutes after irradiation, b: cell fixation at 1 hour after irradiation, c: cell fixation at 4 hours after irradiation, d: cell fixation at 48 hours after irradiation. Photons = γ-rays. Plots show mean (dots) with standard deviations (error bars), lines show linear regression lines. Values are baseline subtracted. Four wells per condition, on average 440 nuclei per well were imaged.

**References**

- Parisi, A., Beltran, C.J. and Furutani, K.M., 2022. The Mayo Clinic Florida microdosimetric kinetic model of clonogenic survival: formalism and first benchmark against in vitro and in silico data. Physics in Medicine & Biology, 67(18), p.185013.
- Sato, T., Iwamoto, Y., Hashimoto, S., Ogawa, T., Furuta, T., Abe, S.I., Kai, T., Tsai, P.E., Matsuda, N., Iwase, H. and Shigyo, N., 2018. Features of particle and heavy ion transport code system (PHITS) version 3.02. Journal of Nuclear Science and Technology, 55(6), pp.684-690.
